# Gastric perforation secondary to T-cell lymphoma

**DOI:** 10.3332/ecancer.2023.1498

**Published:** 2023-01-19

**Authors:** Joaquin Fernandez-Alberti, Matías Mihura Irribarra, Agustín Rancati, Nicolas Panzardi, Maria Florencia Cora, Daniela Speisky, Daniel Enrique Pirchi

**Affiliations:** 1 General Surgery Department, Hospital Británico de Buenos Aires, Perdriel 74, CABA, 1280, Buenos Aires, Argentina; 2 Anatomic Pathology Department, Hospital Británico de Buenos Aires, Perdriel 74, CABA, 1280, Buenos Aires, Argentina; a https://orcid.org/0000-0002-1827-7361; b https://orcid.org/0000-0001-7131-7951; c https://orcid.org/0000-0003-3506-8120; d https://orcid.org/0000-0002-1092-1132; e https://orcid.org/0000-0001-5794-7084; f https://orcid.org/0000-0002-7353-0470

**Keywords:** primary gastric lymphoma, T-cell lymphoma, spontaneous perforation, gastrectomy

## Abstract

**Introduction:**

Malignant primary lymphoma represents only 1%–5% of all gastric tumours. Spontaneous gastric perforation in the absence of chemotherapy in these cases is extremely rare. The vast majority of primary gastric lymphomas have a B-cell phenotype that originates from mucosa-associated lymphoid tissue and primary gastric lymphomas with a T-cell phenotype are rarely reported. This report describes a case of a primary gastric T-cell malignant lymphoma associated to spontaneous perforation and peritonitis.

**Case presentation:**

An 80-year-old woman referring 24 hours of abdominal pain associated to cognitive impairment consulted to our Emergency Department. Her past medical history revealed smoking, hypothyroidism, dilated cardiomyopathy, hypertension, celiac disease with poor adherence to gluten-free diet and a Non-Hodgkin T cell lymphoma associated to enteropathy in 2010. At physical examination, she presented with tachycardia, hypotension and abdominal tenderness. Lab test revealed low red cell count and an abdomen computed tomography scan showed pneumoperitoneum secondary to a large gastric perforation located in the anterior wall of the antrum. Urgent surgery was performed. At exploratory laparoscopy, a 5 cm perforation of the anterior wall of prepyloric antrum was observed associated to a 4-quadrant peritonitis. Conversion to open surgery was decided to perform an open antrectomy and Billroth II gastro-jejunostomy. The patient was transferred to ICU after surgery under mechanical respiratory assistance for closed monitoring but evolved with a cardiogenic shock and deceased on the first postoperative day. The final histopathological and immunohistochemical analysis reported enteropathy-associated T-cell lymphoma of gastric localisation with concomitant celiac disease.

**Discussion:**

We present a rare case of a patient with a history of celiac disease who developed a gastric perforation secondary to an enteropathy-associated T-cell lymphoma of gastric localisation. To the best of authors’ knowledge, there have been reported less than 30 cases of spontaneous perforation of gastric lymphoma in the absence of chemotherapy in the last 35 years. Malignant gastric lymphoma, accounting only for 1% of primary gastric malignancies, is usually a diffuse large B-cell lymphoma. Incidence of perforation of gastric lymphomas in patients receiving chemotherapy rounds 0.9%–1.1%. However, it is a rare condition in patients not receiving chemotherapy.

**Conclusion:**

This is a rare case of a patient with an enteropathy-associated T-cell lymphoma of gastric localisation, who developed a spontaneous gastric perforation in the absence of chemotherapy. Despite it is a rare condition, it must be suspected in patients with a history of lymphoma in the context of acute abdominal pain.

## Introduction

The gastrointestinal tract is the most common extra-nodal site of non-Hodgkin’s lymphoma, accounting for approximately 4%–18% of cases in Western countries. Malignant primary lymphoma represents only 1%–5% of all gastric tumours [1]. The vast majority of primary gastric lymphomas have a B-cell phenotype that originates from mucosa-associated lymphoid tissue and primary gastric lymphoma with a T-cell phenotype is rarely reported [2].

Even though the exact incidence and risk of lymphoma in celiac patients is still a debated issue [3], it is well-known that this condition increases the risk of developing enteropathy-associated T-cell lymphomas compared to the general population [4, 5]. Nonetheless, it appears clear that the development of complications in celiac patients, although infrequent, is an event that negatively impacts on patient survival [6]. In fact, the occurrence of intestinal perforation in a patient affected by celiac disease should lead to suspicion of lymphoma.

Perforation of a gastric malignant lymphoma during chemotherapy is a well-known event. However, the incidence is not high, occurring in about 0.9%–1.1% of cases [7, 8]. Furthermore, spontaneous perforation of a gastric lymphoma in the absence of chemotherapy is extremely rare [9] having been reported in less than 30 cases in the last 35 years [10–12]. This report describes a case of primary gastric T-cell malignant lymphoma associated to spontaneous perforation and peritonitis.

## Case presentation

An 80-year-old woman consulted to our emergency department presenting abdominal pain of 24 hours of onset associated to cognitive impairment. Her past medical history revealed smoking, hypothyroidism, dilated cardiomyopathy, hypertension, celiac disease with low adherence to gluten-free diet and a Non-Hodgkin T-cell lymphoma associated to enteropathy diagnosed in 2010 secondary to an enterectomy performed in context of an acute abdominal pain at another medical centre. After the resection, the patient underwent chemotherapy [R-CHOP regimen: an immunochemotherapy regimen consisting of rituximab, cyclophosphamide, hydroxydaunorubicin hydrochloride (doxorubicin hydrochloride), vincristine (Oncovin) and prednisone used to treat both indolent and aggressive forms of non-Hodgkin lymphoma].

**Figure 1.**
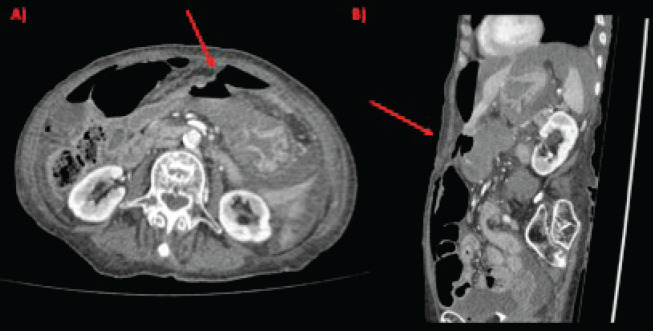
CT scan showing gastric perforation (red arrow) and pneumoperitoneum in (a): axial section and (b): sagittal section.

**Figure 2.**
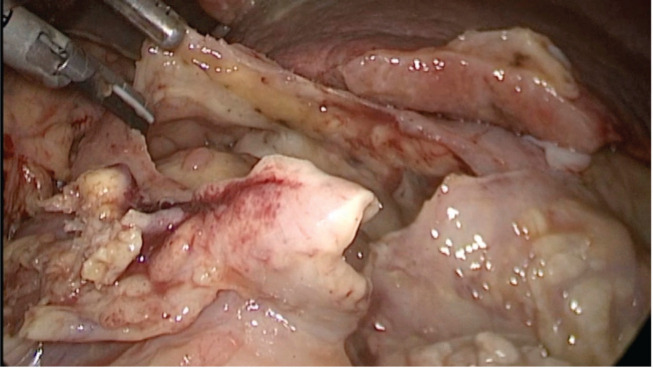
Exploratory laparoscopy evidencing massive perforation of the anterior wall of the gastric antrum.

Physical examination evidenced hypotension (90/50 mm Hg), tachycardia, cognitive impairment and abdominal tenderness. Laboratory tests showed only low red blood cell count (25% for normal values of 36%–46%) with no other abnormalities. Fluid replacement and vital support were initiated with immediate haemodynamic improvement. An abdominal computed tomography (CT) scan was then requested, evidencing pneumoperitoneum and gastric mucosal enhancement with a clear stop in continuity in the greater curvature prior to the antropyloric junction with no other abnormal findings (Figure 1a and b). Immediate intravenous antibiotic treatment and urgent surgery were conducted. Exploratory laparoscopy was performed evidencing a 4-quadrant peritonitis secondary to a 5 cm perforation at the antropyloric junction caused by a necrotic malignant tumour (Figure 2). Due to the patient’s haemodynamic instability, conversion to open surgery was decided to perform an antrectomy and Billroth II gastro-jejunostomy. Full inspection of the gastrointestinal tract was performed during surgery, without any abnormal findings. Nasogastric tube was placed for gastric decompression as well as a trans-anastomotic tube for enteral nutrition. Two drains were left as witnesses of intraabomdinal conditions. The patient was transferred to the ICU in critical condition requiring mechanical respiratory assistance and vasoactive drugs for support. On the first postoperative day, the patient developed a cardiogenic shock that did not respond to resuscitation manoeuvres, causing the patient’s death.

**Figure 3.**
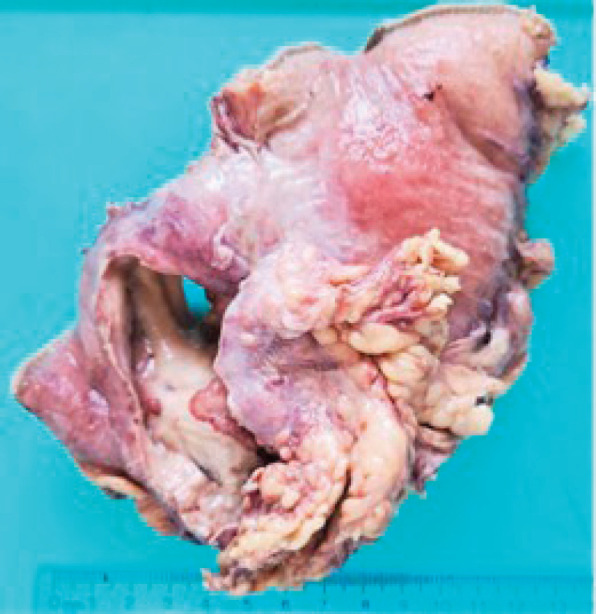
Resected specimen evidencing an ulcerated whitish tumour of 5.6 × 3.8 cm. The lesion involved and perforated the gastric wall with extension into the serosa.

Grossly, the resected specimen showed an ulcerated whitish tumour of 5.6 × 3.8 cm. The lesion involved and perforated the gastric wall with extension into the serosa (Figure 3).

**Figure 4.**
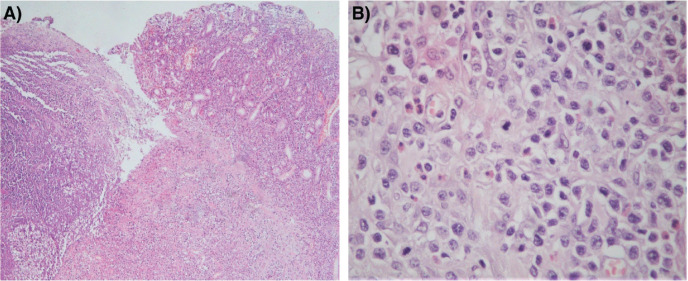
(a): Histological section (H&E – Magnification 40×). The left portion of the image shows mucosal ulceration with a dense tumour cell infiltration underneath. The right half shows adjacent mucosa with expansion of lamina propria by medium-to-large size moderately pleomorphic cells. (b): Histological section (H&E – Magnification 400×) Neoplastic T cells form a dense, moderately pleomorphic infiltrate, medium to large in size with round or angled nuclei and prominent nucleoli. Frequent mitotic figures are observed. There is a component of inflammatory cells, including histiocytes and eosinophils.

The histological sections of material, embedded in paraffin and coloured with haematoxylin and eosin, showed a neoplastic proliferation of diffuse medium to large cells with abundant cytoplasm and round or angulated nuclei, accompanied by a pronounced and polymorphic inflammatory background and extensive areas of necrosis (Figure 4). Neoplastic cells formed an ulcerating mucosal mass with transmural infiltration of the stomach.

**Figure 5.**
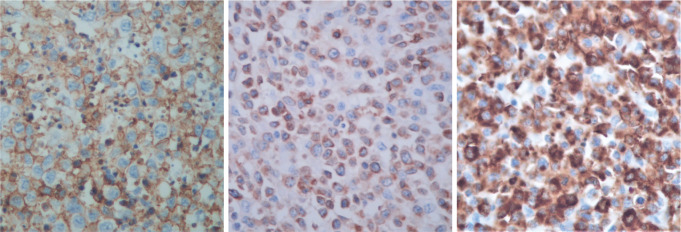
Immunostaining results.

The immunohistological techniques were carried out on histological sections of 3 microns by means of an automated system in accordance with the manufacturer’s guidelines (Benchmark XT, ULTRA). Atypical cells showed positivity for CD45, CD3, CD7 and CD8, according with T-Cell lineage. Staining for CD30, CD4, CD5, CD56, anaplastic lymphoma kinase-1 and keratin cocktail (AE1AE3) as well as B-cell differentiation markers (CD20, PAX5 and CD138) was found to be negative (Figure 5).

There was no expression of Epstein–Barr virus latent membrane protein 1.

The histopathological and immunohistochemical findings, together with the patient’s medical history, allowed the diagnosis of enteropathy-associated T-cell lymphoma of gastric localisation in a patient with celiac disease.

## Discussion

We present a rare case of a patient with a history of celiac disease who developed a gastric perforation secondary to an enteropathy-associated T-cell lymphoma of gastric localisation. To the best of authors’ knowledge, there have been reported less than 30 cases of spontaneous perforation of gastric lymphoma in the absence of chemotherapy in the last 35 years.

Gastrointestinal lymphoma is an uncommon disease but it is the most frequent site of extranodal lymphoma and it is almost exclusively of the non-Hodgkin type. Primary gastrointestinal lymphoma most commonly involves the stomach but can affect any part of the gastrointestinal tract from the oesophagus to the rectum. Risk factors for the development of a gastrointestinal lymphoma include *Helicobacter pylori* infection, immunosuppression after solid organ transplantation, celiac disease, inflammatory bowel disease and human immunodeficiency virus infection. Malignant gastric lymphoma, accounting only for 1% of primary gastric malignancies, is usually a diffuse large B-cell lymphoma.

It is relatively well known that malignant gastric perforations often take place during chemotherapy secondary to the weakening of the gastric tissue associated with rapid tumour necrosis, tumour lysis and exuberant granulation [10]. Maisey *et al* [7] and Yoshino *et al* [8] reported that perforation of gastric lymphomas in patients receiving chemotherapy occurs in about 0.9%–1.1% of cases. However, they are even less frequent in patients not receiving chemotherapy. Moreover, Fukuda *et al* [13] found that less than 5% of malignant gastric lymphomas may complicate with perforation.

In recent years, the standard treatment for aggressive gastric lymphoma has shifted from surgery to chemotherapy. Despite this, there are some authors like Ohkura *et al* [9] who suggest that dose reduction of chemotherapy or even gastrectomy should be considered if a giant ulcer and necrosis on the ulcer floor are present on the upper gastrointestinal endoscopy because of the risk of gastric perforation. Unfortunately, probably due to the COVID-19 pandemic, our patient lost follow-up and consulted directly to our emergency department when her gastric lymphoma was already perforated.

## Conclusion

This is a rare case of a patient with an enteropathy-associated T-cell lymphoma of gastric localisation who developed a spontaneous gastric perforation in the absence of chemotherapy. Despite the fact that it is a rare condition, it must be suspected in patients with a history of lymphoma in the context of acute abdominal pain.

## Conflicts of interest

None declared.

## Funding

The authors have not declared a specific grant for this research from any funding agency in the public, commercial or not-for-profit sectors.

## Authors’ contributions

All authors contributed to the study conception and design. Material preparation, data collection and analysis were performed by Drs Joaquin Fernandez-Alberti, Matías Mihura Irribarra, Agustín Rancati, Nicolás Panzardi, Maria Florencia Cora, Daniela Speisky and Daniel Enrique Pirchi. The first draft of the manuscript was written by Joaquín Fernandez-Alberti and all authors commented on previous versions of the manuscript. All authors read and approved the final manuscript.
